# Clinically Feasible and Accurate View Classification of Echocardiographic Images Using Deep Learning

**DOI:** 10.3390/biom10050665

**Published:** 2020-04-25

**Authors:** Kenya Kusunose, Akihiro Haga, Mizuki Inoue, Daiju Fukuda, Hirotsugu Yamada, Masataka Sata

**Affiliations:** 1 Department of Cardiovascular Medicine, Tokushima University Hospital, Tokushima 770-8503, Japan; daiju.fukuda@tokushima-u.ac.jp (D.F.); yamadah@tokushima-u.ac.jp (H.Y.); masataka.sata@tokushima-u.ac.jp (M.S.); 2 Department of Medical Image Informatics, Graduate School of Biomedical Sciences, Tokushima University, Tokushima 770-8503, Japan; haga@tokushima-u.ac.jp (A.H.); circulation@outlook.com (M.I.)

**Keywords:** echocardiography, artificial intelligence, view classification

## Abstract

A proper echocardiographic study requires several video clips recorded from different acquisition angles for observation of the complex cardiac anatomy. However, these video clips are not necessarily labeled in a database. Identification of the acquired view becomes the first step of analyzing an echocardiogram. Currently, there is no consensus whether the mislabeled samples can be used to create a feasible clinical prediction model of ejection fraction (EF). The aim of this study was to test two types of input methods for the classification of images, and to test the accuracy of the prediction model for EF in a learning database containing mislabeled images that were not checked by observers. We enrolled 340 patients with five standard views (long axis, short axis, 3-chamber view, 4-chamber view and 2-chamber view) and 10 images in a cycle, used for training a convolutional neural network to classify views (total 17,000 labeled images). All DICOM images were rigidly registered and rescaled into a reference image to fit the size of echocardiographic images. We employed 5-fold cross validation to examine model performance. We tested models trained by two types of data, averaged images and 10 selected images. Our best model (from 10 selected images) classified video views with 98.1% overall test accuracy in the independent cohort. In our view classification model, 1.9% of the images were mislabeled. To determine if this 98.1% accuracy was acceptable for creating the clinical prediction model using echocardiographic data, we tested the prediction model for EF using learning data with a 1.9% error rate. The accuracy of the prediction model for EF was warranted, even with training data containing 1.9% mislabeled images. The CNN algorithm can classify images into five standard views in a clinical setting. Our results suggest that this approach may provide a clinically feasible accuracy level of view classification for the analysis of echocardiographic data.

## 1. Introduction

In the modern era, artificial intelligence (AI) utilizing deep learning (DL) has been used as a developing tool to assist diagnosis in the medical field [1,2,3,4,5,6]. AI may also have significant potential in the assessment, diagnosis and prognosis of cardiovascular disease [7,8,9,10]. Conventional machine learning usually requires derivation of predefined features in the input image [11]. In contrast, DL automatically estimates results from the image without the need to predefine specific imaging features [1,12]. In addition, the deep layers of the convolutional neural network are able to extract detailed low-level information from the original image and combine these to form higher order structural information, enabling the identification of complex entities from the images. This technique may be useful in analyzing echocardiographic findings of various heart diseases [13,14,15,16].

Echocardiographic images include several video clips for assessment of the complex cardiac structure and require standard views to properly diagnose cardiovascular diseases. Determination of the view is the first step in analyzing an echocardiogram. However, echocardiographic data are generally labeled inadequately and there are differences in image properties in the dataset. Thus, accurate identification of individual echocardiographic views is required in order to develop a feasible AI algorithm for the assessment of cardiovascular diseases. Previous reports have tested the performance of a view classification AI model using echocardiographic images [17,18,19]. However, these studies are based on samples from a narrow range of left ventricular ejection fraction (EF) (e.g., Madani et al. study: EF < 55%: 20%–22% patients) [18]. In addition, there is no consensus whether the mislabeled samples can be used to create a feasible clinical prediction model of ejection fraction (EF). The aim of this study was to test two types of input methods for the classification of images, and to test the accuracy of the prediction model for EF in a learning database containing mislabeled images that were not checked by observers.

## 2. Methods

### 2.1. Study Population

We enrolled 340 patients (Table 1) with 5 standard views (long axis, short axis, 3-chamber view, 4-chamber view and 2-chamber view) and 10 images in a cycle, for training a classification model using a convolutional neural network (total 17,000 labeled images). To compensate for the small size of the dataset, we sampled the patients so that their cardiac function was evenly distributed over a wide left ventricular ejection fraction (LVEF) range (40 patients had LVEF = 10%–20%, 50 patients had LVEF = 21%–30%, 50 patients had LVEF = 31%–40%, 50 patients had LVEF = 41%–50%, 50 patients had LVEF = 51%–60%, 50 patients had LVEF = 61%–70% and 50 patients had LVEF = 71%–80%). We selected cases with good or adequate acoustic detail, determined by visualization of the LV walls and endocardium, testing the DL algorithm on echocardiographic images gathered on machines of two different vendors (EPIQ and Vivid E9/E95).

To overcome the issue of vendor dependency and heterogeneous EF distribution, we gathered a separate validation group of 189 patients who were referred to our echocardiographic laboratory. The validation group included no images from the training group. These views were obtained using various ultrasound machines (EPIQ and iE33; Philips Healthcare, Amsterdam, The Netherlands; Vivid E9/95; GE Healthcare, Waukesha, WI; Preirus; Hitachi, Tokyo, Japan; SSA-770A; Canon Medical, Otawara, Japan) with varied image qualities. The Institutional Review Board of the Tokushima University Hospital approved the study protocol (No. 3217-3).

### 2.2. Import Data of Echocardiography

**Figure 1:**
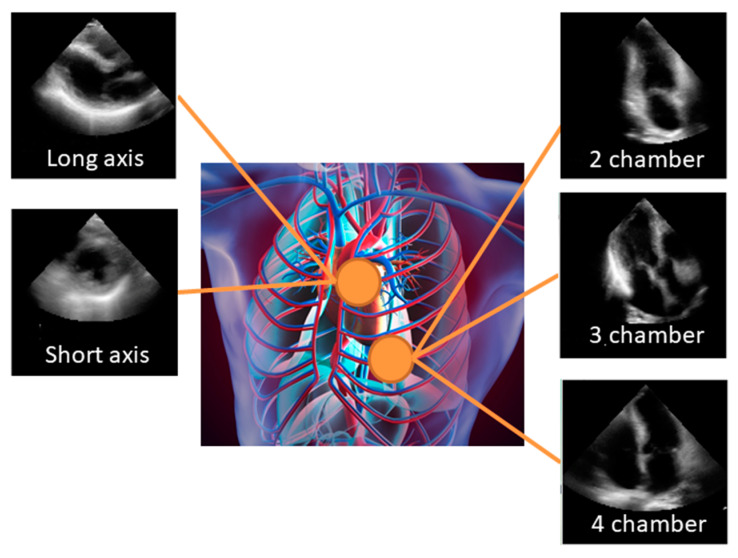
Standard views: The apical 2-chamber (AP2), apical 4-chamber (AP4), apical 3-chamber (AP3), parasternal long axis (PLAX), and parasternal short axis (PSAX) views were stored digitally for playback and analysis. Echocardiographic images shown here are the average of 10 consecutive images.

All echocardiographic measurements were obtained according to the recommendations [20]. The apical 2-chamber (AP2), apical 4-chamber (AP4), apical 3-chamber (AP3), parasternal long axis (PLAX), and parasternal short axis (PSAX) views were stored digitally for playback and analysis (Figure 1). Each case contained cardiac ultrasound images from the AP2, AP4, AP3 PLAX and PSAX. All DICOM images were rigidly registered and rescaled into a reference image to fit the size of echocardiographic images. The images were cut and down sampled to 18.07 × 18.07 cm^2^ with 120 × 120 pixels in monochrome. Simultaneously, we removed the metadata presented in the periphery of these images by adjusting the color window. We sampled the clips in sequential order. To adjust for differences in frame rate and heart rate, we selected 10 equally spaced images per 1 cardiac cycle with a semi-automatic heartbeat analysis algorithm. Thus, we analyzed 2D+t data, with input images made of 120×120 pixels. All data were divided into 5 groups. Of these groups, 4 were used for training and validation to create a model, and the remainder was used in the testing of this model. Namely, the 17,000 views were split into 5 groups of 3,400 views. Then, 5-fold cross validation was employed to examine model performance. The model’s generality was also validated by the independently-gathered data from 189 patients. For the view classification model, we designed two types of input data. The first is images averaged over 10 images in sequential order. The second is the original 10 selected images and the class prediction was done by averaging over 10 images.

### 2.3. Deep Learning Model

**Figure 2:**
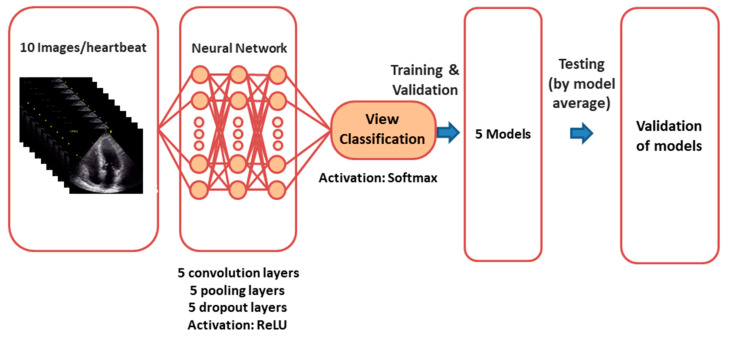
Neural network for view classification: We designed and trained convolutional neural network models to recognize 5 different standard echocardiographic views.

The overall process of model creation is shown in Figure 2. Classification of views was accomplished by a CNN algorithm. We designed and trained three CNN models to recognize 5 standard echocardiographic views. The first model used the averaged image over time as an input. The input image was 2D with a size of 120 × 120 pixels. The second model used 10 selected images with a size of 120 × 120 pixels, trained independently, and the averaged probability (of each of the 5 predicted classes) was employed in the prediction phase. We also checked the performance using other models. The details of trials using other models such as “ImageNet” are listed in Supplementary Materials.

We trained three CNN architectures. The cross-entropy error function was used as a loss function to be reduced. The number of iterations (epochs) was set at 50 from the behavior of validation loss. The weights with the minimum validation loss were stored. Thus, we created five weight sets for 5-fold cross validation. For the test using the independent cohort (189 patients), the probability calculated by each weight set was averaged, and the view with the maximum averaged probability was classified. Model training was performed on a graphics processing unit (GeForce GTX 1080 Ti, NVIDIA, Santa Clara, California, USA). In all cases, the Adam optimizer with the default parameters was used for training. DL was performed with the Python 3.6 programming language with Keras 2.1.5.

### 2.4. Statistical Analysis and Evaluation

The diagnostic performance of the CNN algorithm was evaluated using a contingency table, which is tabulated with the class giving a maximum probability. We calculated the weighted kappa to assess the accuracy of this model. In order to test the accuracy of the prediction model for EF in the learning database containing mislabeled images that were not checked by observers, we used the previously developed model for the prediction of LVEF [21]. Agreement between LVEF based on CNN and reference LVEF was expressed using Pearson’s correlation coefficients. Statistical analysis was performed using standard statistical software packages (SPSS software 21; SPSS Inc, Chicago, IL, USA, and MedCalc Software 19; Mariakerke, Belgium). Statistical significance was defined by *p* < 0.05.

## 3. Results

### 3.1. View Classification

**Figure 3:**
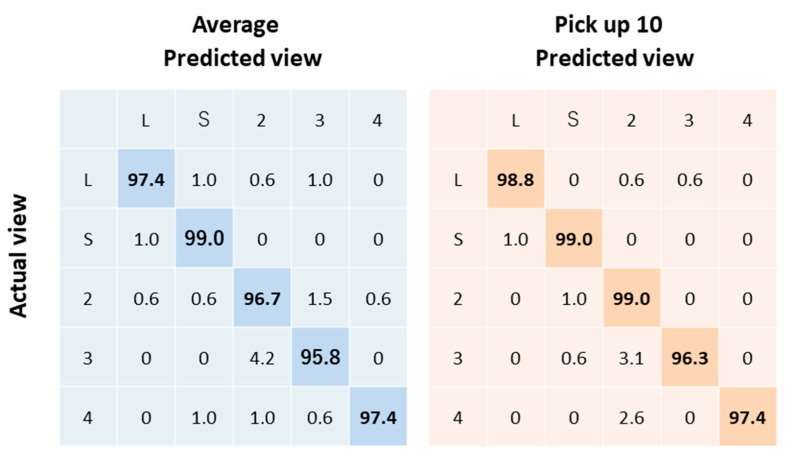
Echocardiogram view classification by deep-learning model: Actual view labels are on the *y*-axis, and neural network-predicted view labels are on the *x*-axis by view category for video classification.

We tested models trained by two sets of data consisting of averaged images, and 10 selected images in the independent cohort (n = 189). Our best model (from 10 selected images) classified videos into five views with 98.1% overall test accuracy (Figure 3). The weighted kappa was 0.98 ± 0.005 (95%CI: 0.971 to 0.989).

### 3.2. Misclassification

**Figure 4:**
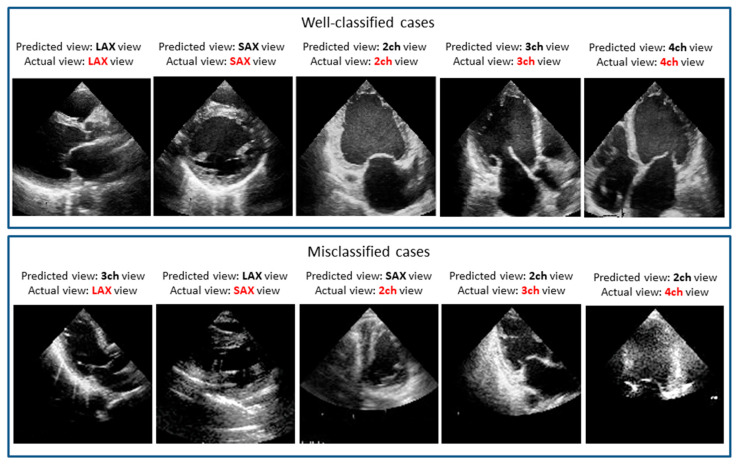
Well-classified and misclassified cases: For the misclassified cases, it seems to be difficult even for expert observers to determine the accurate view.

Because a softmax function was employed as an activation function at the final layer, this component in the output vector can be regarded as the probability of giving the corresponding view. In our prediction model, the probability was further averaged over the five models created in the 5-fold cross validation. With the maximum probability given by this averaging, almost all cases were classified successfully. However, small portions were still misclassified. We manually checked these misclassification cases. The similarity of images explains the misclassification of these images, which most often involved views that look similar to human eyes. These include adjacent views in echocardiographic acquisition, where a slight difference in the angle of the sonographer’s wrist can change the view, resulting in the confusion of an apical three chamber view for an apical two chamber view or an apical four chamber view. Figure 4 shows examples of misclassified cases. Interestingly, the misclassified cases seem to be difficult to determine even by expert observers.

### 3.3. Acceptable Error Rates

In our view classification model, 1.9% of the images were mislabeled. To determine if this 98.1% accuracy level was acceptable for creating a clinical prediction model using echocardiographic data, we tested the prediction model for EF (see reference [21] for details) using learning data with a 1.9% error rate. There were good correlations from two datasets (with no errors and a 1.9% error rate) between reference LVEF and estimated LVEF (r = 0.80 from the dataset with a 1.9% error rate vs. r = 0.82 from dataset with no errors). Even with training data containing 1.9% mislabeled images, the accuracy of the echocardiogram prediction model for LVEF was warranted.

To check the relation between the number of mislabeled samples and the performance of the prediction model for LVEF, we added the accuracy of this prediction model in the database with 0.5% and 1.5% mislabeled images. There were good correlations between reference LVEF and estimated LVEF using the database with 0 (r = 0.82), 0.5% (r = 0.82), 1.5% (r = 0.78) and 1.9% (r = 0.80) mislabeled images. There was no statistical difference among the correlations (compared p > 0.05). Thus, we concluded that a mislabeling rate within 1.9% (resulting from our view classification model) did not significantly affect the accuracy of the EF prediction model.

## 4. Discussion

View classification is a key step for the interpretation of echocardiographic images in a clinical setting. We tested a deep-learning model that correctly classified conventional echocardiographic data to different views in the test cohort. The test cohort data were gathered from consecutive clinical datasets acquired for clinical purposes, from patients with a wide range of ages, sizes, and hemodynamics. Moreover, even after the use of learning data with a 0.5% error rate, the accuracy of the echocardiogram prediction model for LVEF was warranted. This model is a feasible image classification method. In summary, our study showed 1) 98.1% overall test accuracy of a CNN algorithm in the independent cohort, and 2) 99.5% of samples need to be correctly labeled to create a feasible clinical prediction model of EF. A previous study (Madani et al.) [18] used samples based on a narrow range of EF (Madani et al. study: EF < 55%: 20–22% patients. Our study: EF < 55%: 60% patients). Compared to this previous study, our study included patients with a wide variety of systolic functions, recorded by machines of various vendors.

### 4.1. Deep Learning for Echocardiography

We established two new findings. The first was the good agreement for classification of videos. Video analysis can be a complex undertaking that involves many trivial tasks, such as frame-to-frame color variation and object tracking. Effective view classification can make this process more efficient and cost-effective, reducing coding and training time. Second, the accuracy of the echocardiogram prediction model for LVEF from the database with a 0.5% error rate was good. Some papers showed the excellent accuracy of the view classification AI model (around 91.7% to 98.9% for view classification) using echocardiographic images [17,18,19]. Our analysis adds to this by demonstrating the good performance of a CNN algorithm and that this CNN algorithm can be used to develop the echocardiographic prediction model for EF.

In addition, we checked the appropriate layer number and its node number at the initial stage of the present study with the selected cohort. As these numbers were increased, the prediction accuracy tended to be increased. In the present model, five layers with 64 to 128 nodes yielded the best performance. We also checked the optimal cropping size of the input images by comparing among the pixel sizes of 100, 120, and 200 in space (see Supplementary Materials). According to these tests, we decided to use a pixel size of 120 in the classification. Based on this checking, we believe our model is fit for application to echocardiographic images.

### 4.2. Limitations

The number of patients was relatively limited. DL algorithms may require thousands of images in some cases (e.g., when the variety of data is immense, as in the ImageNet challenge). Using good-quality data with appropriate labeling can reduce the number of cases needed to build a DL model. In our analysis, the DL diagnostic accuracy was excellent (98.1% overall test accuracy) in the separate validation cohort (consecutive patients with varied image qualities at our laboratory), strongly supporting the generalization performance of the present approach. We did not explore the acceptable error rate to create the prediction model for EF in detail (for example, from 0% to 10% by 0.5% steps). However, the accuracy of our view classification model was enough to create the prediction model for EF. Further study will be planned to explore the acceptable error rate. We did not compare the CNN models with other ML models in this study. In our previous analysis on prediction of LVEF, we used a feature extraction method, consisting of cross correlation and optical flow. In the results, the feature extraction method was not suitable to predict the LVEF using echocardiographic images compared with CNN models (feature extraction method: r = 0.58 vs. CNN models: r = 0.92, *p* < 0.001). Thus, we decided to apply the CNN model for echocardiographic view classification in this study. Our results confirm in principle that CNN may be very effective in the view classification, but larger numbers of patients should be evaluated to measure the efficacy of automatic diagnosis systems in a clinical setting.

## 5. Conclusions

Our results suggest that this approach may provide clinically acceptable and accurate view classification in the analysis of echocardiographic data.

## Figures and Tables

**Table 1:** Baseline characteristics of the study population.

	Control
Number	340
Age, years	66 ± 14
Male, %	58
Ischemic Cardiomyopathy, %	48
Heart rate, bpm	77 ± 16
LVEDVi, ml/m^2^	74 (53–105)
LVESVi, ml/m^2^	40 (20–74)
LVEF, %	45 (29–62)

Data are presented as number of patients (percentage), mean ± SD or median (interquartile range). Abbreviations: LVEDVi, left ventricular end diastolic volume index; LVESVi, left ventricular end systolic volume index; WMSI, wall motion score index; LVEF, left ventricular ejection fraction.

## Supplementary Materials

The following are available online at https://www.mdpi.com/2218-273X/10/5/665/s1, Supplemental Data.
