# Supplementary material for: Clinically Feasible and Accurate View Classification of Echocardiographic Images Using Deep Learning
Source: Biomolecules. 2020 Apr 25;10(5):665. doi: 10.3390/biom10050665 (PMC7277840; doi:10.3390/biom10050665)
Supplement: Supplementary file 1 [file biomolecules-10-00665-s001.pdf]

Supplemental Data

Clinically Feasible and Accurate View Classification of Echocardiographic Images using Deep Learning

1. Details of the classification model used

A detailed structure of the deep neural network (DNN) model used in this study is shown in Fig. S1, where the network comprises a total of 5 convolutional layers of N, $2N$, $2N$, $2N$, and N filters with kernel sizes of 3 × 3, and 5 pooling layers of kernel size 2 × 2 were applied. A series of 2 fully connected layers—with 512, and 5 units—were calculated in the final layer. Activation function used in all activation layers is Rectified Linear Unit (Relu) except for the last activation, where the softmax function is employed. Number of the filters in the layer is controlled by N, and the result with N = 64 was shown in the manuscript. This value was determined by the grid search in the range of [16, 256] in the initial stage of the present work (see the next section).

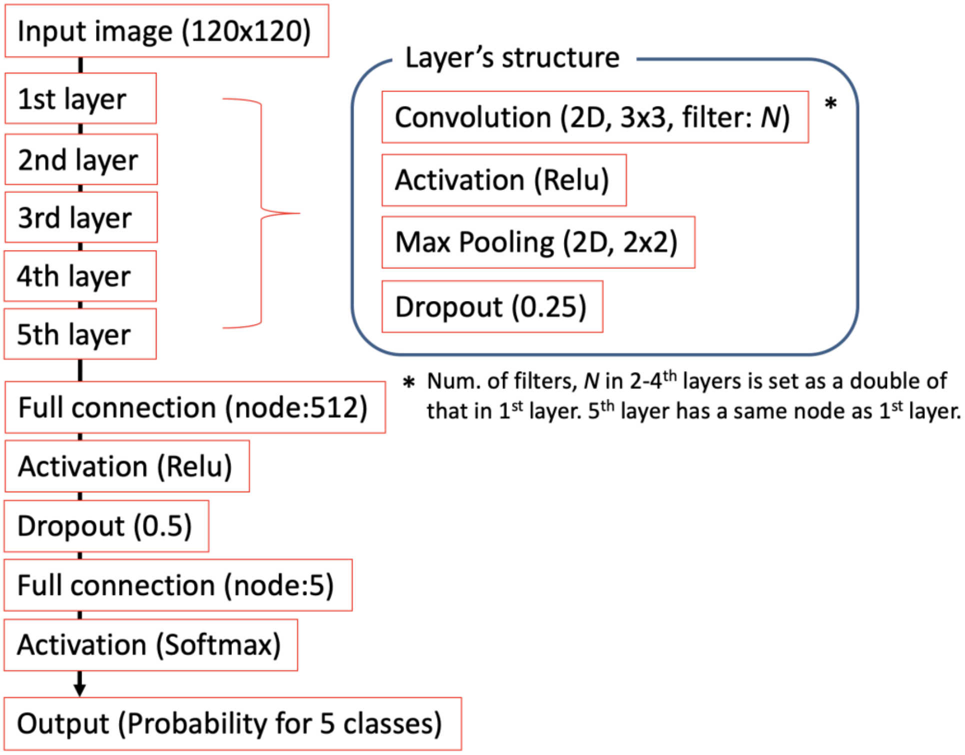

Fig. S1: Proposed deep neural network model.

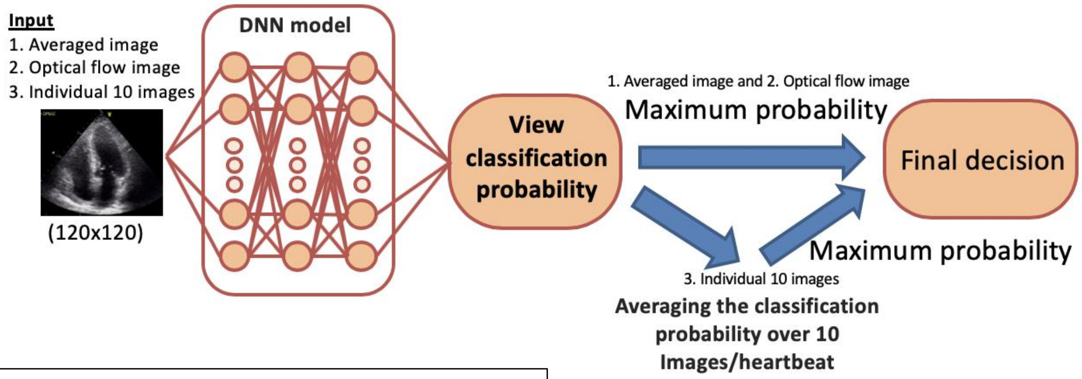

Fig. S2: Proposed classification model.

With this DNN, the view classification model was constructed as shown in Fig. S2. The details are also found in the manuscript.

2. Model optimization

We checked the appropriate layer number and its filter number at the initial stage of the present study with the selected cohort. In this stage, the averaged image was employed. The cohort included 340 patients, which was divided as (204, 68, 68) for (train, validation, test), respectively. In the training, the cross-entropy error function was employed as the loss function. We used the ADAM optimizer with the default parameters except for an initial learning rate of 0.0005 and batch size of 5. The number of the epochs was set as 50. The model weights were stored when the validation loss was minimized. The accuracy of the view classification for the test cohort was used in the evaluation. The result was shown in Fig. S3, where the 3D bar graph indicating the accuracy is plotted for various numbers of the layer and the filter.

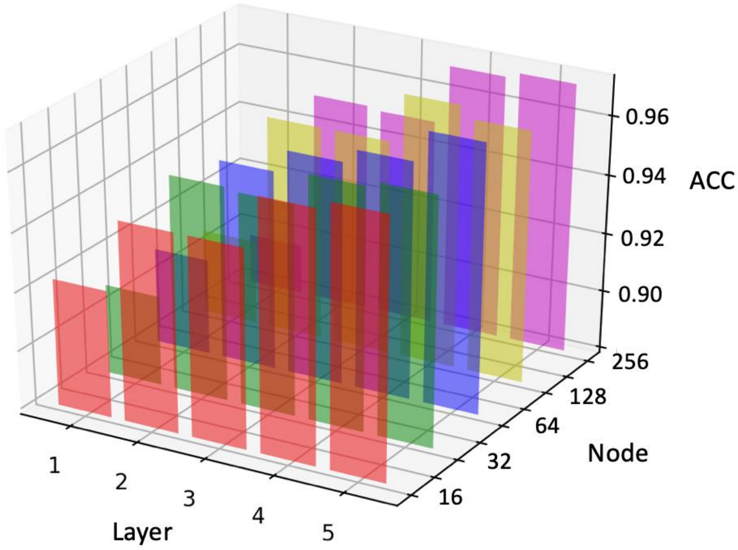

Fig. S3: Accuracy in test cohort (changing the number of layers and filters).

It was found that 5 layers with N = 64 filters yielded the best performance. Using this model, the dependency of the input image size was also checked and shown in Fig.S4.

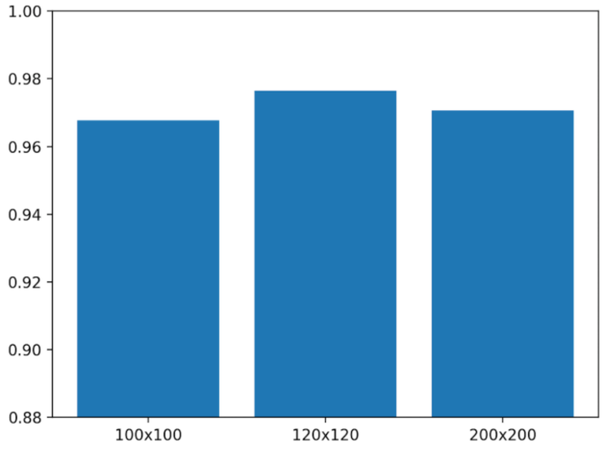

Fig. S4: Accuracy in test cohort (changing the image size). The DNN model was 5 layers with *N* = 64 filters.

3. Other models

We examined DNN models other than the model described in the manuscript; two dimensional (2D) CNN models by utilizing the RGB channel with three images. The model is a similar framework of Fig. S1, though the input image has three channels. For this, we selected 3 out of 10 sequential images. Here, the image pair giving the minimum of the cross correlation was first

selected and the rest was selected from the middle of the image pair. The selected 3 images were assigned into the RGB channel (hereafter, the model using RGB images is called as "RGB model"). The training data were augmented by sliding the initial image of a cardiac cycle in each data. Other than this original model, for comparison, we also examined well-established models indicated in https://keras.io/applications/. In this supplement, we show the result from "ResNet50" which is one of the representative models in ImageNet.

In Fig. S5, the view classification accuracy for test cohort (68 cases) are indicated. In this comparison, the cohort included 340 patients, which was divided as (204, 68, 68) for (train, validation, test). The RGB model and the ResNet50 model gives a comparable result with that in the 10-images model.

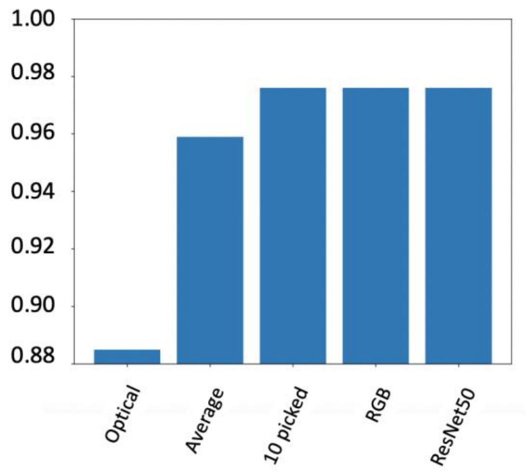

Fig. S5: Accuracy in test cohort using various models
